# Preliminary report on osteochondrosis in cattle in the north-western parts of South Africa

**DOI:** 10.4102/ojvr.v83i1.1083

**Published:** 2016-07-27

**Authors:** Leon Prozesky, Johan Neser, Heinz Meissner, Kenneth Botha, Lubbe Jacobs, Craig Shepstone, Hannes Viljoen, Hinner Köster, Chris de Brouwer, Jan van Zyl, Gerjan van der Veen

**Affiliations:** 1 Department of Paraclinical Sciences, University of Pretoria, South Africa; 2 Lubern Animal Feeds, Hartswater, South Africa; 3 Animal Nutrition and Health, Centurion, South Africa; 4 Kaonna Investments (Pty) Ltd, Pretoria, South Africa; 5 Department of Agriculture, North West Province, South Africa; 6 Farmer North West Province

## Abstract

The north-western part of South Africa, in particular, is well known for mineral imbalances. Aphosphorosis, resulting in rickets and osteomalacia, received a lot of attention at the turn of the nineteenth century (1882–1912). This was followed in 1997 by research on Vryburg hepatosis, another area-specific mineral imbalance–related disease in young calves reared on manganese-rich soil derived from the weathering of dolomitic (carbonate) rock formations. In 1982, a totally new syndrome (osteochondrosis) manifested in, amongst others, areas in South Africa where aphosphorosis was rife. Osteochondrosis was also identified in the south-western parts of Namibia as well as southern Botswana and other areas in South Africa. Osteochondrosis has a multifactorial aetiology and this study focused on the role of minerals, particularly phosphorus, in the development of the disease. A significant improvement in the clinical signs in experimental animals and a reduction of osteochondrosis occurred on farms where animals received bioavailable trace minerals and phosphorus as part of a balanced lick. An increase in the occurrence of the disease on farms during severe drought conditions in 2012–2013 prompted researchers to investigate the possible role of chronic metabolic acidosis in the pathogenesis of the disease.

## Introduction

Since the 1800s, the north-western parts of South Africa, particularly the North West and Northern Cape Provinces, have been known as an area characterised by aphosphorosis (‘stiff sickness’) in cattle (Theiler 1912; Theiler *et al*. 1927), caused by a phosphorus deficiency that resulted in osteomalacia. Affected animals often developed botulism because of the consumption of bones contaminated with *Clostridium botulinum* (Theiler, Du Toit & Malan 1937; Theiler *et al*. 1927). The bone meal and coarse salt licks introduced to prevent osteomalacia and botulism seemed to have resolved the osteomalacia or rickets syndrome in the north-western parts of the country. However, in 1982, farmers and local veterinarians started reporting lameness accompanied by swelling of the stifle joint in cattle.

Initially, the lameness seemed to affect only a small number of show animals and it was also seen in Phase D tested bulls, but the incidence steadily increased, and in 2004, farmers reported an incidence that ranged from 2% to 20%. In some instances up to 40% of animals were affected. The syndrome was found to be present in cattle of all age groups and classes (both commercial and non-commercial), and apparently in most breeds of cattle farmed in that area. Afrikaner cattle seemed to be more resistant to this condition. The only factor in common amongst affected animals was the geographical area in which they lived.

Necropsies performed on 21 severely affected cattle revealed chronic lesions that enabled a diagnosis of osteochondrosis to be made. Osteochondrosis is a common and important joint disorder that occurs in humans and many animal species, particularly pigs, horses and dogs (Bradley & Dandy 1989). It is defined as a focal disturbance of endochondral ossification and has a multifactorial aetiology (Ekman & Carlsson 1998; Gerber *et al*. 1999; Schenk & Goodnight 1996; Ytrehus, Carlson & Ekman 2007). Whilst existing evidence supports the importance of genetic and conformational factors (Ytrehus *et al*. 2007), a number of possible aetiologies and predisposing factors, such as over-nutrition, rapid growth, genetics (experimentally proven in pigs [Hittmeier *et al*. 2006; Ytrehus *et al*. 2004]), ischaemia, excess dietary calcium, hormonal influences and trauma, have been proposed (Trostel, McLaughlin & Pool 2002; Wooderd 1997). In human medicine, osteochondrosis has been defined as an idiopathic condition characterised by disorderliness of endochondral ossification (Siffert 1981).

As early as 1978, it was suggested that the pathophysiology of osteochondrosis is essentially the same in all species, including humans (Olsson 1987; Olsson & Reiland 1978). Although formation of fragile cartilage, failure of chondrocyte differentiation, subchondral bone necrosis and failure of blood supply to the growth cartilage have all been proposed as an initial step in the pathogenesis, the recent literature strongly supports failure of blood supply to growth cartilage as being the most likely (Ytrehus *et al*. 2004, 2007). Localised failure of endochondral ossification ensues, which typically results in necrosis of affected cartilage. It can involve the physis or the articular–epiphyseal cartilage (Hill, Sutton & Thompson 1998; Reiland 1978; Trostel *et al*. 2002). Mild trauma to the necrotic cartilage may result in cleft formation of the affected cartilage, with release of cartilage degeneration products into the joint causing a synovitis evident clinically as joint effusion and pain with lameness (Trostel *et al*. 2002). In pigs, the lesion is slightly different in that there is no evidence of necrosis of cartilage, but rather a focal failure of mineralisation of hypertrophic cartilage cells (Wooderd 1997). In dogs, osteochondrosis lesions are typically bilateral (Trostel *et al*. 2002).

Osteochondrosis in cattle is found in all types of husbandry systems, including feedlots (Davies & Munro 1999; Heinola *et al*. 2006; Jensen *et al*. 1981; Reiland *et al*. 1978), pure-bred beef (Dutra, Carlsten & Ekman 1999), dairy (Trostle *et al*. 1997) and animals grazing rangelands (Hill *et al*. 1998).

Cattle affected with this disorder develop effusions in the weight-bearing joints, in particular the femoro-tibial (stifle) joint, associated with inflammation and pain, causing lameness of varying degrees. As a result, animals are unable to walk long distances for grazing, have decreased feed intake, have decreased milk production and a loss in body condition, and bulls have decreased mating ability (Persson, Soderquist & Ekman 2007). Animals are often eventually slaughtered as a result of severe lameness and loss of condition.

Because, in the North West Province, outbreak cattle of multiple breeds were affected, and the area is characterised by mineral imbalances, samples of liver and rib were subjected to mineral analysis together with samples taken from healthy control cattle and water samples from the affected area. A limited feeding trial on seven cattle of the same group as the necropsied cattle was undertaken. Based on the results obtained, various studies were subsequently undertaken to examine the possible role of mineral imbalances in the syndrome, the details of which will be published elsewhere. The study describes the pathology of the lesions observed at necropsy on which the diagnosis of osteochondrosis is based. The other important syndromes caused by mineral imbalances in the same area are discussed, and the results of the mineral analysis and pilot feeding trial are briefly summarised.

## Materials and methods

During June 2004, 28 clinically affected female adult animals (2–7 years old), representing various breeds, were donated by farmers from the North West Province to the University of Pretoria for research purposes. The animals showed lameness and articular swelling that was most prominent in the stifle joint. From the time of arrival, animals were fed only good quality *Eragrostis* sp. hay without any mineral supplement (lick).

### Pathological examinations

Necropsies were conducted on 21 animals (5 animals 2–3 years old and 16 animals 4–6 years old). All the joints of the limbs were opened and examined, as well as multiple vertebral joints, and lesions were described and photographed. Bone samples were taken for histopathological examination, and bone and liver samples were taken for mineral analysis (to be described in detail elsewhere).

### Feeding trial

The remaining seven animals (three 2–3 years old and four 4–6 years old) with less severe lameness and swelling of the stifle joints were fed on a commercial production supplement (Maxitech, AFGRI) at a recommended daily intake of 1.5 kg per animal per day for a period of 3 months, from August to October 2004.

An additional supply of a commercial 6% phosphorus supplement (Sukrafos) containing macro- and micro-minerals was also fed for the first month at a daily intake of 80 g per animal per day. *Eragrostis* sp. hay was supplied *ad libitum.* Water supplied by the local municipality was also freely available. The animals were slaughtered and necropsied at the end of the trial.

## Results

### Pathology

**FIGURE 1:**
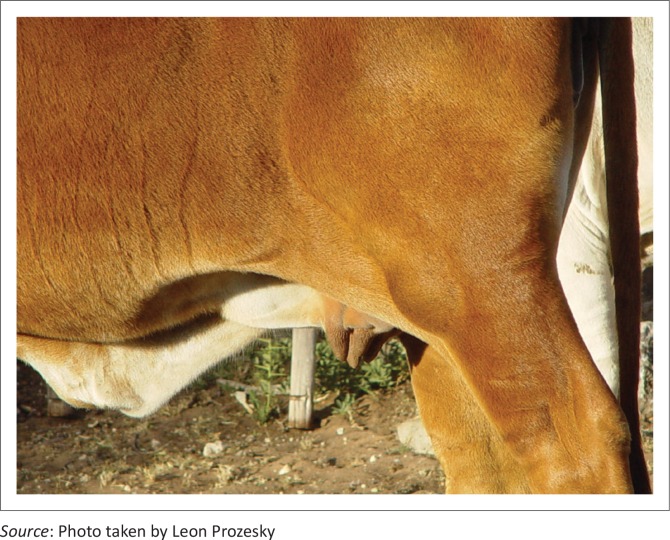
Joint effusion of the femoro-tibial joint in a clinically affected animal with osteochondrosis.

Lesions involving the articular cartilage were present in all the animals in various joints (including the atlanto-axial, tibio-tarsal, scapular-humeral and femoral-pelvic joints), but were consistently most prominent in the femoro-tibial joints, often evident clinically as a joint effusion (Figure 1). The macroscopic and microscopic lesions associated with osteochondrosis are well documented, and the lesions noted in the experimental cattle did not differ from those reported (Hill *et al*. 1998; Reiland 1978; Ytrehus *et al*. 2007). For the purpose of this article, the lesions are briefly summarised.

**FIGURE 2:**
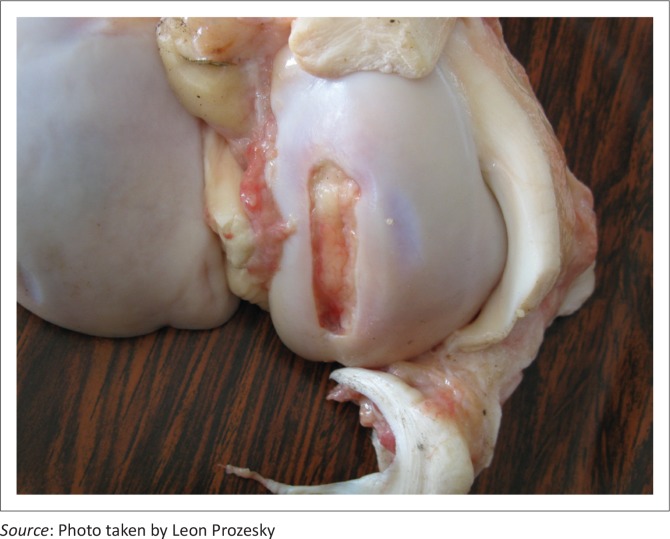
Irregular crater in the right femur condyle.

**FIGURE 3:**
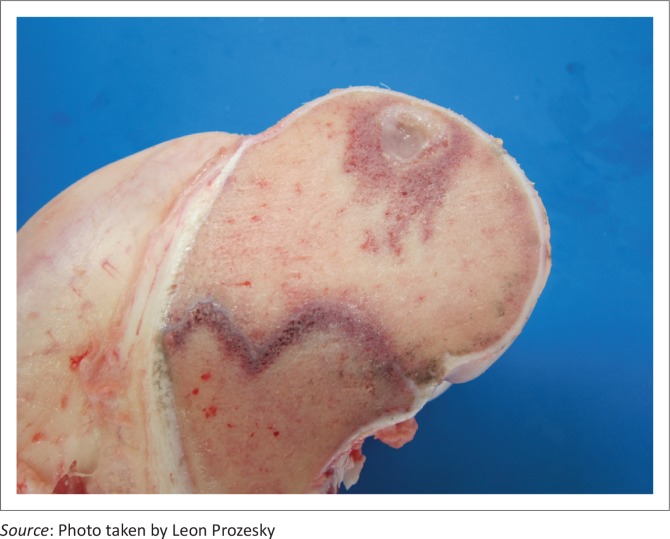
Subchondral cysts containing necrotic material and surrounded by well-vascularised fibrous connective tissue.

**FIGURE 4:**
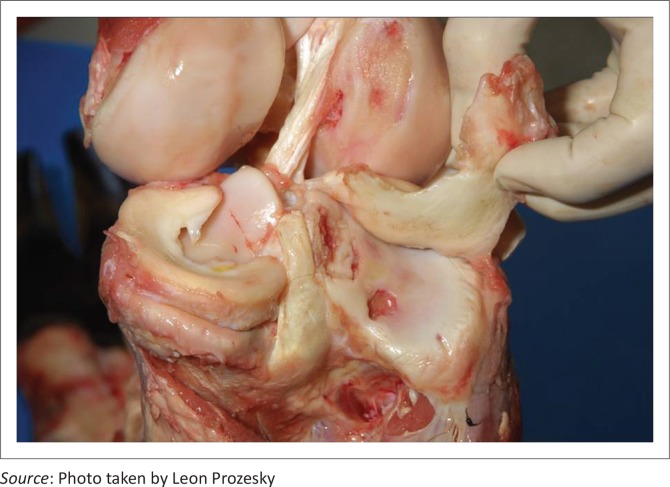
Severe degenerative chondral lesions extending to the menisci.

There was notable variation in the extent of the lesions. The opposing articular cartilage surfaces lacked the normal lustre and had a mildly eroded appearance. In the stifle joints, lesions on the medial and lateral condyles ranged from shallow clefts to several converging fissures, to multifocal to coalescing ulcerations forming grooves and irregular craters in the thickened cartilage (Figure 2) that sometimes formed subchondral cysts containing necrotic material. The cysts were surrounded by well-vascularised fibrous connective tissue (Figure 3). Severe degenerative lesions extended to the menisci (Figure 4).

**FIGURE 5:**
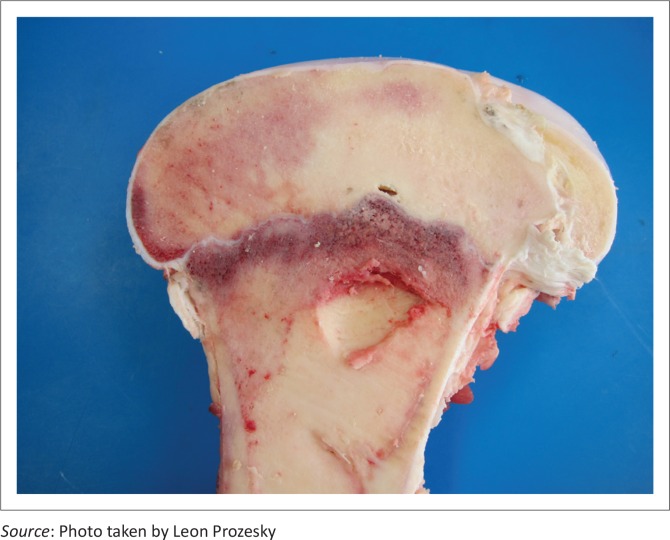
Abnormal trabecular pattern of the growth plate was noted in young animals.

**FIGURE 6:**
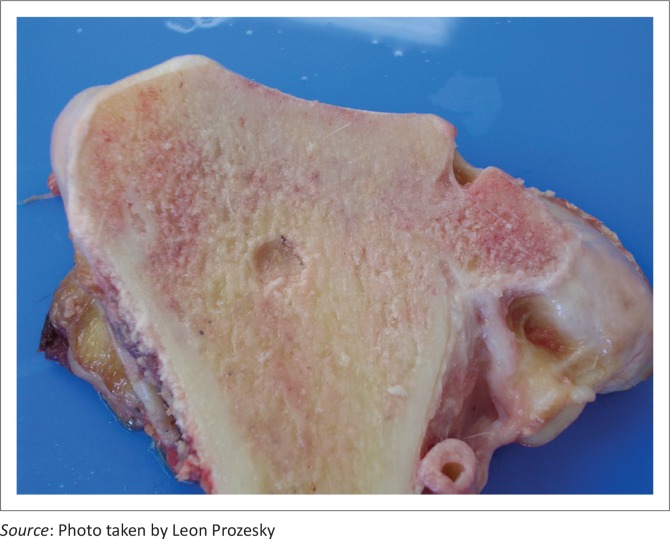
Cyst at the level of the growth plate (older animal).

Ulceration of the articular cartilage often resulted in an exposed, irregular connective tissue base. Abnormal trabecular pattern of the growth plate was noted in young animals, whereas in some older animals, lesions in the growth plate indicative of endochondral failure were characterised by the presence of a cyst approximately 10 mm – 15 mm in diameter at the level of the growth plate (Figures 5 and 6). The joint spaces contained recently detached flaps of degenerative cartilage or osteochondral bodies. In all the animals, the joint capsules showed mild to moderate fibrous thickening, with hyperplasia of the *stratum synoviale*. Joints contained copious amounts of synovial fluid, which appeared macroscopically normal.

**FIGURE 7:**
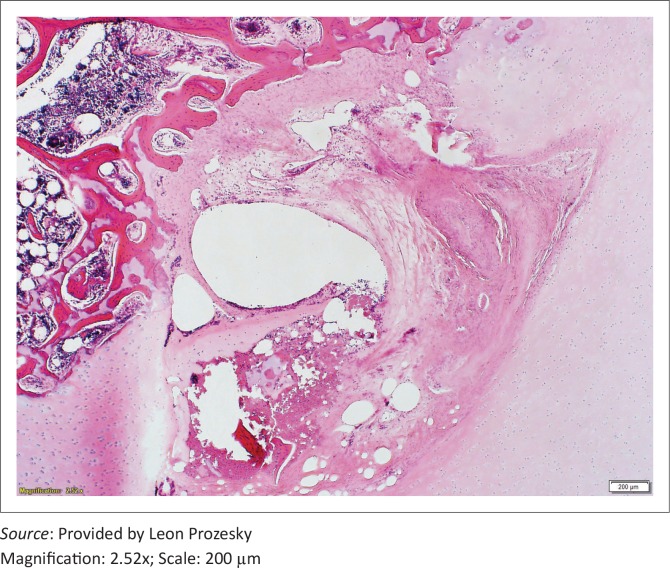
Extensive necrosis of the articular–epiphyseal cartilage complex extending to the subchondral bone.

Representative lesions on the distal femur were selected to demonstrate the histopathological lesions. Lesions were noted in the entire articular–epiphyseal cartilage complex, involving both the avascular articular cartilage and the vascular epiphyseal cartilage, and extended into the underlying subchondral bone (Figure 7). Multifocal necrosis was present in the vascularised epiphyseal cartilage, and the latter was covered by a thick necrotic cartilage layer. Clefts extended from the articular surface to the subchondral bone and contained multifocal necrosis and well-vascularised connective tissue (granulation tissue) that sometimes extended into the ulcerated articular cartilage complex.

### Feeding trial

**FIGURE 8:**
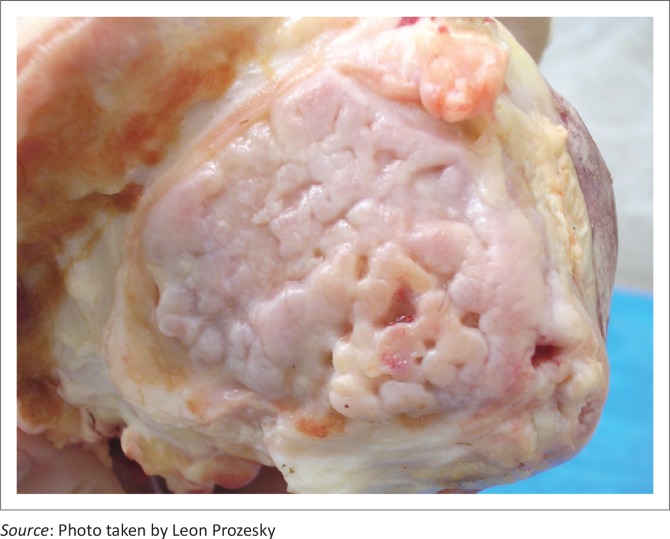
Note irregular fibrous articular cartilage covering the femur condyle.

After a period of 3 months it was agreed by several observers, including two experienced farmers from the North West Province, that a significant improvement in the degree of external swelling of the most severely affected stifle joints had occurred and that this was accompanied clinically by a reduction in the degree of lameness, particularly in the younger animals. All the animals also showed an increase in body condition score; at the start of the trial, they had a score of 2 and all improved to a score of 3. Body mass also increased by an average of approximately 43 kg per animal. At necropsy, joint fluid samples were collected and tested negative for bacteria and mycoplasmas. Lesions in the joints and bones were similar to those described in the slaughtered animals, except that in the younger animals, the degenerative hyaline cartilage was replaced by irregular fibrous cartilage (Figure 8).

## Discussion

The pathological lesions confirmed a diagnosis of osteochondrosis. The current study confirmed that failure of endochondral ossification resulting in necrosis of either the physis or the articular–epiphyseal cartilage was central in the pathogenesis of the lesions, as proposed by Ytrehus *et al*. (2004) and Ytrehus *et al*. (2007). To the best of our knowledge, the high incidence of osteochondrosis in cattle in South Africa since 1982, particularly in, although not confined to, the north-western parts of the country, represents the largest outbreak of this condition ever recorded in cattle. It has had a major impact on beef production in the affected areas. It is therefore important that the cause of the problem should be established in order to determine how to manage it.

Even though the clinical signs and macro- and microscopic lesions associated with osteochondrosis are well documented, to a large extent the aetiology remains controversial. Problems associated with the study of osteochondrosis include, amongst others, the multifactorial aetiology proposed for the condition, a lack of parameters to measure the improvement in affected animals (e.g. following a change in the diet because of the relatively slow regenerative capacity of cartilage and bone, which are the main tissues affected) and the difficulty in producing clinical cases experimentally. Whilst strong evidence has been adduced for breed and conformation being influential in the development of osteochondrosis (Ytrehus *et al*. 2007), it seems probable that other factors may play a role in the present outbreak. Although apparent sparing of the Afrikaner breed of cattle as well as a probable conformational link have been observed in the outbreak, the wide range of breeds and ages affected and the geographical distribution of the outbreak are suggestive of additional contributory factors, some of which, in particular nutritional factors, may be of major importance.

From a geological point of view, the north-western parts of South Africa, the south-western parts of Namibia, as well as southern Botswana where osteochondrosis was reported are characterised by the presence of superficial dolomitic (carbonate) rock formations. The area also has a history of well-documented syndromes in cattle that are demonstrably because of mineral deficiencies or imbalance. The best known of these is osteomalacia resulting from aphosphorosis, described by Theiler (1912), which also results in lameness and became known as ‘stiff-sickness’ (*stijfziekte*). It was therefore important to establish whether the outbreak that started in 1982 was a manifestation of aphosphorosis or a new condition.

Stiff sickness and botulism (*lamziekte*) were diseases of cattle commonly found in the north-western parts of South Africa and assumed to be two different manifestations of the same disease. It was soon suspected, and later demonstrated, to be linked to a phosphate deficiency (Theiler 1912; Theiler *et al*. 1937), and in 1919 Theiler discovered and later proved that botulism was a disease distinct from osteomalacia and was caused by toxins released from *C. botulinum* present in bones the cattle were ingesting as a result of pica (Theiler *et al*. 1927).

Osteomalacia is essentially defective mineralisation of osteoid in adult animals. Its pathogenesis is very similar to that of rickets in young animals. In rickets, there is abnormal endochondral ossification of the cartilage growth plates as well as defective bone formation at sites of bone remodelling. In osteomalacia, because the growth plates have closed, only sites of bone remodelling are affected. The essential lesion is defective mineralisation of the cartilage matrix of growth plates (rickets) and defective mineralisation of osteoid (both diseases). Osteomalacia and rickets are manifestations of one of a number of possible nutritional abnormalities, viz. aphosphorosis, vitamin D deficiency, calcium deficiency or calcium:phosphorus imbalance (Thompson 2007).

The lesions are variable, and a diagnosis may therefore be difficult. In rickets, the forelimbs are often bowed and the ends of the long bones at the growth plates are enlarged, as are the costochondral junctions. In severe cases of rickets, pathological fractures are common. In osteomalacia, the bones are weaker than normal, resulting in excessive bone matrix deposition where stresses and strains on the bones are greatest. As in rickets, the bones fracture easily. Macroscopically, the cortices of the bones are thin, spongy and soft. Kyphosis or lordosis is often noted. The thorax is often narrowed and flattened, and the sternum may be prominent (Thompson 2007; Wooderd 1997).

Historically, osteomalacia occurred only on certain farms, where it was endemic every year at more or less the same time. Affected animals had a severe craving for bones. Young growing, pregnant animals or cows with calves at foot were most commonly affected, and the condition was most prevalent when the grazing was poor, for example, during times of drought. Clinically affected animals appeared to experience severe pain and showed signs of arthritis and laminitis, and in young animals, signs of rickets were noted. Animals showed a shifting lameness, lying down frequently. They walked with their backs arched and their hind legs tucked up underneath the abdomen and placed their weight on their heels, which resulted in abnormal hoof growth (Theiler 1912).

Pica noted in animals with stiff sickness was proven experimentally to be associated directly with aphosphorosis. The main stimulus attracting phosphorus-deficient cattle to bones is olfactory, with old bones being their first choice. Phosphorus-deficient cattle are not attracted to other animal products such as fat, meat or blood. An intravenous injection of sodium phosphate was found to abolish the cravings for bones within minutes (McDowell 1996). Provision of bone meal and coarse salt licks resolved the problem of osteomalacia and botulism in the north-western parts of the country. Following the outbreak of bovine spongiform encephalopathy in the United Kingdom in 1985, the use of bone meal in South Africa as a feed supplement was banned, but supplementation of animals with a high phosphorus–containing lick (P18) continues to prevent cattle from developing osteomalacia at the research station where the original research on the condition was carried out (C. de Brouwer, pers. comm., 2014).

Vryburg hepatosis is an area-specific mineral imbalance–related disease. The name is derived from the district in the North West Province in South Africa where most of the cases originated and the characteristic histopathological changes were observed in the livers of affected animals (Neser *et al*. 1997). The condition occurs in young calves 7–14 days old reared on manganese-rich soil derived from the weathering of dolomitic (carbonate) rock formations in the Northern Cape and North West Provinces. Affected animals develop severe subacute to chronic cholangiohepatitis with icterus and most untreated animals succumb to the disease. The disease manifests clinically in young calves as geophagia, and affected animals sometimes also lick iron poles. Geophagia is followed by constipation, dehydration and death within 7–10 days. On severely affected farms, 50% – 75% of calves may develop geophagia, with a mortality rate close to 100% in untreated cases. Liver analysis revealed high manganese levels, ranging from 10 ppm to 1800 ppm wet mass (normal range 2 ppm – 3 ppm wet mass). Grazing plants from affected farms had higher levels of manganese and a higher ratio of manganese–to-iron than plants from unaffected farms (Neser *et al*. 1997). Manganese interferes with iron and cobalt absorption from the gastrointestinal tract (Hurley & Keen 1986; Thompson & Valberg 1972). Treatment of calves by injection with commercial iron-dextran compounds and vitamin B_12_ at 1–2 days after birth and repeated at 14 days of age has a significant preventative effect. Furthermore, in endemic areas, geophagia can be prevented by rearing calves in enclosures covered with a thick layer of dung. Vryburg hepatosis was experimentally induced by dosing a calf and two lambs with relatively high levels of manganese oxide (Elsenbroek & Neser 2002; Neser *et al*. 1997).

The diagnosis of osteochondrosis indicated that the current outbreak likely had a different aetiology from osteomalacia. A survey of affected farms revealed that factors associated with the current outbreak included the following: geographical area, breed (fast-growing breeds were more severely affected than slow-growing breeds), sex (male animals were more severely affected than female animals), age (weaner’s were more affected than adult animals), conformation of the animals (cattle with structural bone deformities, e.g. straight hocks were more at risk), nutritional status of the animals, management systems (calving throughout the year compared to fixed calving seasons) and ongoing selection pressures on animals (shorter calving intervals and heavier weaning weights). It was, however, unclear whether any of these were the primary cause of the condition or whether they merely exacerbated it. That the conditions were linked to the area was strengthened when enquiries further afield revealed that the condition was also present in certain geologically similar parts of Namibia and Botswana.

Given the apparent geological link and the fact that an association between nutrition and cattle diseases has been demonstrated in the area, possible involvement of mineral imbalances warranted further investigation, although the involvement of dietary factors was not supported by evidence presented in a recent review (Ytrehus *et al*. 2007). Nevertheless, osteochondrosis has been associated with a copper deficiency in several species, including deer, bison and horses. The lesions are caused either by a primary copper deficiency or by exposure to factors that inhibit copper absorption or metabolism (e.g. zinc, cadmium or inorganic sulphates) (Hurtig *et al*. 1993). These authors further suggested a relationship between low copper intake in fast-growing horses, inferior collagen quality, biomechanically weak cartilage and bone, and lesions of osteochondrosis dissecans. Osteochondrosis associated with a copper deficiency was also reported in New Zealand in young wapiti cross red deer hybrids (Audigé *et al*. 1995; Thompson *et al*. 1994; Wilson & Grace 2001). However, there was no evidence of copper deficiency in the current study.

Davies and Munro (1999) described an outbreak of osteochondrosis in bull beef cattle following failure to provide dietary mineral and vitamin supplementation. Analysis of the metacarpal bone from two bulls revealed adequate magnesium, phosphorus and bone ash, but a slightly low calcium concentration. The vitamin A concentration was also low. Dietary analysis suggested inadequate calcium, sodium and copper intake and mild deficiency of vitamins A, D and E. A balanced mineral and vitamin supplement was added to the diet when it became clear that the supplement had been omitted. A gradual clinical improvement was seen in the majority of the animals, and after 2–3 weeks, the growth rate and coat quality had improved significantly. This outbreak provides evidence that in some cases a mineral and vitamin imbalance is a likely contributing factor to the development of osteochondrosis in growing cattle.

Calcium deficiency, with a distorted calcium-to-phosphate ratio, was associated with an outbreak of osteoarthritis in fattening bulls that was probably osteochondrosis. Calcium deficiency caused more serious lesions in the age group 5–8 months than in the age group 12–18 months. Osteoarthritis lesions occurred in more than 80% of the animals with a calcium-deficient diet (Heinola *et al*. 2006). Similarly, other authors have reported osteochondrosis-type lesions in feedlots and bull stations where the feed given was thought to be one of the causative factors (Jensen *et al*. 1981; Reiland *et al*. 1978; Trostle *et al*. 1998; Weisbrode *et al*. 1982).

The results of the preliminary investigation using samples of liver and rib from affected cattle as well as from cattle from an unaffected herd within the area of the outbreak and an unaffected herd outside the outbreak area revealed that the cattle had low magnesium levels and some of the cattle had low manganese and/or phosphorus levels at the time of testing. Water samples collected from 27 affected farms revealed high levels of bromine in all samples, with nickel, lead and selenium often in elevated concentrations. The very high water hardness values suggest that scaling and poor palatability may pose some problems, whilst contributions to acid–base balance aspects may also be relevant. It is, however, realised that mineral analysis of liver, bone and water samples from farms where osteochondrosis is rife must be analysed on an ongoing basis to compare results during different times of the year, and particularly during periods of drought, which has a significant influence on the composition of the water. However, preliminary observations including the feeding trial indicate that the primary cause of osteochondrosis in southern Africa may be a deficiency or imbalance in mineral intake.

## Conclusion

Extensive on-farm trials after the completion of this study have confirmed that supplementation with balanced minerals, bioavailable phosphorus and vitamins has a significant impact on preventing and curing the condition in the field. Commercial mineral supplements have been formulated for use in affected areas. An upsurge of the condition during a drought in 2012/2013 has implicated metabolic acidosis as a possible complicating factor, as described in previous studies (Barzel & Jowsey 1969; Bushinsky 2001; Green & Kleeman 1991; Kraut *et al*. 1986; Sutton, Wong & Dirks 1997). Acid–base balance is a state of equilibrium between acidity and alkalinity of the body fluids, also called hydrogen ion balance. The body’s acid–base balance is normally tightly regulated by buffering agents and mechanisms related to the respiratory, renal and skeletal systems, keeping the arterial blood pH between 7.38 and 7.42 (Cunningham & Klein 2007). Investigations into this aspect are being pursued. These studies will be described in detail in future publications.
